# Post-Radiotherapy Carotid Stenosis in a Patient With Head and Neck Cancer: A Case Report

**DOI:** 10.7759/cureus.87537

**Published:** 2025-07-08

**Authors:** Imran Ali Reshi, Irshad Rashid, Kirtan Soni, Linda A Mbah, Preeti Yadav, Jeffrin John Varghese, Mansi Singh

**Affiliations:** 1 Internal Medicine, Government Medical College Srinagar, Srinagar, IND; 2 Internal Medicine, Gujarat Medical Education and Research Society (GMERS) Medical College, Gandhinagar, IND; 3 Primary Care, Veterans Affairs, Fort Wayne, USA; 4 General Physician, Grant Government Medical College, Mumbai, IND; 5 Internal Medicine, Government Medical College Thiruvananthapuram, Thiruvananthapuram, IND; 6 Medicine, O. O. Bogomolets National Medical University, Kyiv, UKR

**Keywords:** cervix cancer, chemoradiotherapy, internal carotid artery stenosis, metastatic squamous cell carcinoma, vascular complications

## Abstract

This case report presents a 68-year-old female with a complex medical history including metastatic squamous cell carcinoma of unknown primary site, treated with excisional biopsy and definitive chemoradiotherapy (CRT), and surgically treated stage 0 cervical cancer. The clinical course includes comprehensive treatment for squamous cell carcinoma involving the lymph nodes of the head and neck region, including chemoradiotherapy and concurrent cisplatin chemotherapy. The patient displayed the presentation of severe proximal right internal carotid artery (ICA) stenosis, picked up during a carotid duplex examination. Diagnostic findings reveal new severe proximal right ICA stenosis compared to prior assessments. This case highlights the importance of continued monitoring of long-term vascular complications post-management of patients who have undergone chemoradiation.

## Introduction

Carotid artery stenosis (CAS) is a recognized consequence of atherosclerotic vascular disease and a significant contributor to the risk of stroke and transient ischemic attacks (TIAs), accounting for approximately 10-15% of cases. Epidemiological studies estimate that CAS affects about 5%-10% of adults over the age of 65, with prevalence increasing with age and risk factors such as hypertension, smoking, and hyperlipidemia [1,2]. It most commonly affects the proximal internal carotid artery and the distal portion of the common carotid artery. While traditional carotid atherosclerosis is linked to conventional cardiovascular risk factors, stenosis resulting from prior radiotherapy represents a distinct clinical entity. Histopathological studies have shown that radiation-induced carotid lesions are typically more fibrotic and exhibit less inflammation compared to those caused by standard atherosclerotic processes [2]. Moreover, radiation-associated stenoses are often characterized by long, segmental plaques located in unusual areas and tend to progress more aggressively [3]. Prompt identification and management are crucial to lowering the risk of cardiovascular complications, which are already elevated in individuals with a history of radiation exposure [2]. We present a case of a 68-year-old woman who developed carotid artery stenosis nine years post-radiotherapy, detected during a routine check-up.

## Case presentation

We report the case of a 68-year-old woman who was diagnosed with significant stenosis of the proximal right internal carotid artery (ICA) in January 2023, despite having normal carotid duplex ultrasound findings three years earlier, in 2020. Her past medical history includes squamous cell carcinoma of an unknown primary site, which had metastasized to the left cervical lymph nodes. A thorough diagnostic work-up, including nasopharyngoscopy and fiber-optic laryngoscopy, failed to reveal the location of the primary tumor.

**Figure 1:**
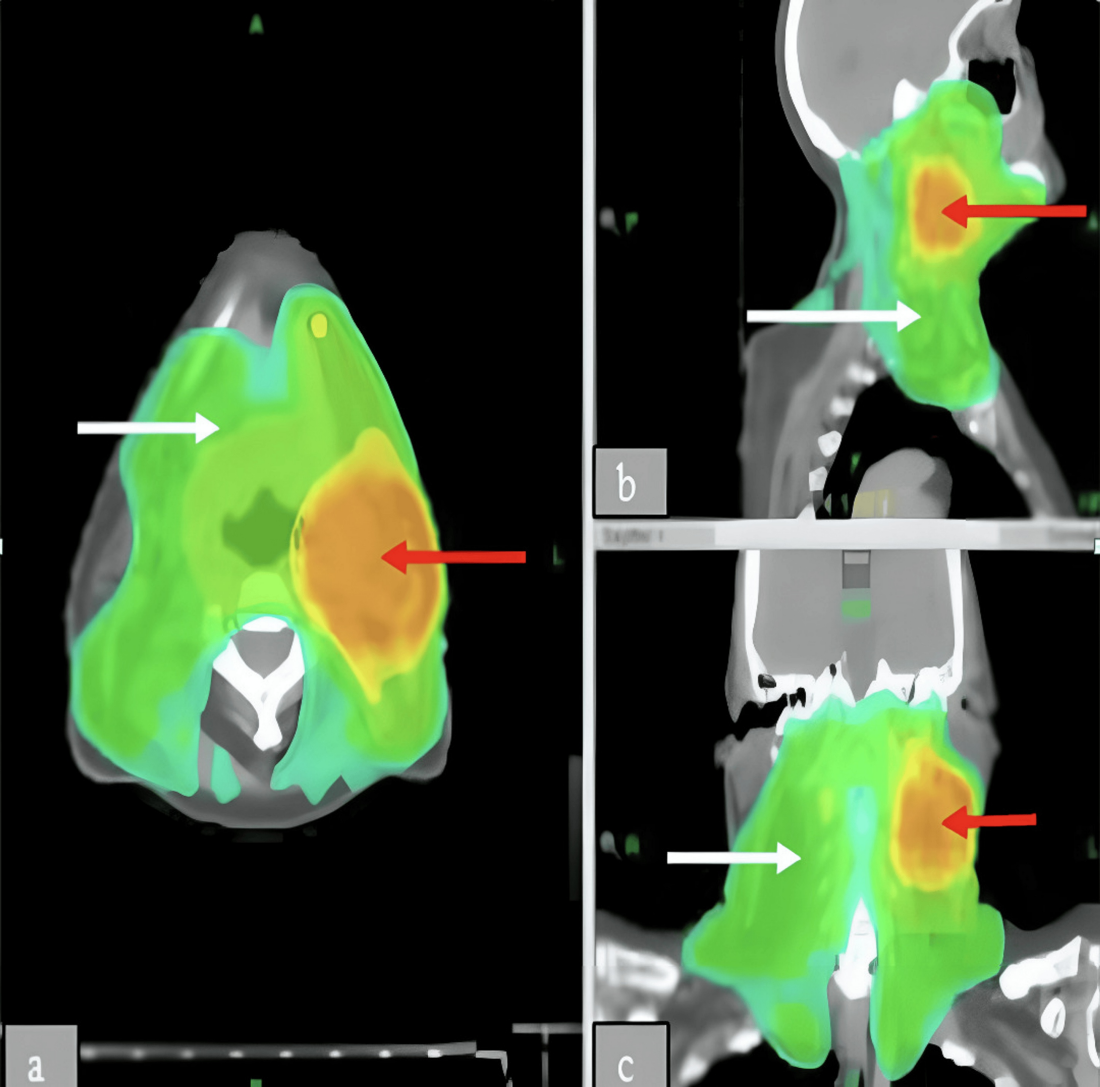
(a-c) Axial IMRT views showing dose distributions to PTVs as part of a head and neck radiation treatment plan. Red arrows show the gross disease. PTV1 (orange/yellow). White arrows show at-risk areas PTV2 and PTV3 (green) IMRT: intensity-modulated radiotherapy, PTV: planning target volumes

She received helical intensity-modulated radiotherapy (IMRT) combined with concurrent cisplatin-based chemotherapy between January and March 2014. The radiotherapy plan consisted of three distinct planning target volumes (PTVs), each receiving a different radiation dose: PTV1 received 66 Gy over 33 fractions, PTV2 received 54.6 Gy across 28 fractions, and PTV3 received 50.4 Gy in 28 fractions. PTV1, marked in orange on Figure 1, represented the high-dose region targeting the known malignancy, specifically the affected lymph nodes. The green-shaded areas in the figure indicate regions receiving lower radiation doses (PTV2 and PTV3), which were included in the plan to address potential microscopic disease spread.

The patient received concurrent chemotherapy with cisplatin at a dosage of 35 mg/m² over seven cycles. In 2015, she underwent a right upper lobectomy for a suspected metastatic lesion; however, histopathological analysis revealed organizing pneumonia, a recognized complication of prior radiotherapy. Her past surgical history also includes a total abdominal hysterectomy with bilateral salpingo-oophorectomy in 2011 for stage 0 cervical carcinoma. Notably, she had no conventional vascular risk factors such as hypertension, diabetes, dyslipidemia, or smoking.

In January 2023, she presented with intermittent episodes of transient visual blurring in the right eye, each lasting only a few seconds. Ophthalmic examination, including fundoscopy, showed no abnormalities of the optic disc. On physical examination, auscultation over the carotid arteries revealed a bruit on the right side. The carotid Doppler ultrasound demonstrated elevated peak systolic velocities in both carotid arteries. Subsequent CT angiography confirmed high-grade occlusion of the right internal carotid artery and mild-to-moderate stenosis of the left common carotid artery. Given the findings, the patient underwent endovascular stenting of the right ICA. Post-procedural imaging confirmed successful recanalization, and she was discharged in stable condition on dual antiplatelet therapy following an uneventful recovery.

## Discussion

The standard approach to managing loco-regionally advanced head and neck squamous cell carcinoma (HNSCC) typically involves either definitive radiotherapy combined with concurrent chemotherapy or surgical resection followed by adjuvant radiotherapy [4]. Among the long-term complications associated with radiation, large vessel pathologies such as carotid artery stenosis (CAS) hold greater clinical importance compared to the more extensively documented microvascular changes. Although there are no universally standardized guidelines, current clinical practice recommends periodic vascular imaging, such as carotid Doppler ultrasound, for patients with a history of head and neck radiotherapy, especially those presenting with neurologic symptoms or vascular risk factors. CAS is a significant risk factor for both ischemic stroke and transient ischemic attacks (TIAs) [5].

Evidence from a review by Carpenter et al. supports the role of radiotherapy (RT) as an independent risk factor for both clinically overt stroke and asymptomatic CAS. Their analysis of head and neck cancer (HNC) patients found that those treated with RT had a higher incidence of cerebrovascular events compared to those treated with surgery alone. Specifically, RT was linked to increased 10-year and 15-year risks of stroke and fatal stroke, respectively. Furthermore, cross-sectional studies have reported a prevalence of asymptomatic CAS in this population ranging from 11.7% at a mean follow-up of 72 months post-RT to as high as 19.8% at 24 months [6].

The underlying pathophysiological mechanisms of radiation-induced CAS involve direct vascular injury. Ionizing radiation leads to endothelial cell apoptosis and senescence, triggering the release of pro-inflammatory cytokines. This cascade results in chronic inflammation, lipid peroxidation, and progressive vascular dysfunction. Monocyte and smooth muscle cell migration into the arterial media occurs in response to oxidized low-density lipoprotein (LDL) accumulation, fostering the development of atherosclerotic plaques. Radiation accelerates atherosclerosis through several pathways: endothelial damage, intimal hyperplasia, medial necrosis, and peri-adventitial fibrosis. Macrophage-mediated release of inflammatory cytokines, including tumor necrosis factor-beta (TNF-β) and transforming growth factor-beta (TGF-β), further perpetuates this inflammatory state [7]. Additionally, cisplatin-based chemotherapy, often administered concurrently with RT, has been shown to exacerbate endothelial injury and may contribute to acute vascular toxicity [8].

Color Doppler ultrasound (CDUS) remains the preferred initial modality for screening carotid artery stenosis (CAS). The B-mode component is particularly useful in assessing plaque morphology and measuring arterial wall thickness. For further anatomical evaluation, CT angiography offers advantages such as superior spatial resolution, rapid acquisition time, and effective visualization of calcified plaques. Magnetic resonance angiography (MRA) is another non-invasive imaging option, considered safe and efficient for assessing vascular stenosis. Multiple studies have demonstrated that MRA provides diagnostic accuracy comparable to that of CT angiography in evaluating CAS [2].

Carotid endarterectomy (CEA) is widely regarded as the gold standard for treating significant carotid stenosis. However, individuals with prior neck radiotherapy present a unique surgical challenge and are generally considered high-risk candidates. Radiation-induced periadventitial fibrosis complicates dissection by obscuring tissue planes and compromising arterial wall integrity. Additionally, radiation-related lesions are often more extensive and may require advanced vascular reconstruction or replacement, thereby increasing perioperative morbidity. The incidence of local wound complications is also higher in this population. Consequently, carotid artery stenting (CAS) is often favored as a less invasive and safer alternative in this context [9].

A meta-analysis by Fokkema et al. compared outcomes between CEA and CAS in patients with a history of head and neck radiation. The study found that CEA was associated with a higher incidence of temporary cranial nerve injury, whereas CAS carried a greater long-term risk of cerebrovascular events and restenosis of 50% or more. Despite these differences, the findings were inconclusive regarding a superior intervention [10].

The economic viability of routine CAS screening depends on disease prevalence. According to the American Society of Neuroimaging, such screening is cost-effective in populations where CAS prevalence is 20% or higher. Based on findings by Carpenter et al., it may be appropriate to initiate screening for asymptomatic CAS in head and neck cancer survivors within 2 to 5 years following completion of radiotherapy [6].

## Conclusions

In conclusion, this case highlights the potential risk of post-radiotherapy carotid stenosis in patients with head and neck cancer, particularly those receiving intensive treatment regimens involving high-dose radiotherapy and concurrent chemotherapy. Long-term surveillance for vascular complications is essential in the management of these patients to facilitate early detection and intervention, thereby minimizing the risk of cerebrovascular events and optimizing long-term outcomes.
